# Paradoxical Hyperreactivity in Bilateral Carotid Stenosis as a Potential Mechanism for Convexity Subarachnoid Hemorrhage: A Case Report

**DOI:** 10.7759/cureus.93118

**Published:** 2025-09-24

**Authors:** Matteo Paolucci, Giacomo Urbinati, Ludovica Migliaccio, Simone Galluzzo, Mauro Gentile, Stefano Merolla, Luana Gentile, Salvatore Isceri, Laura Piccolo, Luigi Simonetti, Andrea Zini

**Affiliations:** 1 Department of Neurology and Stroke Centre, Maggiore Hospital, IRCCS Istituto delle Scienze Neurologiche di Bologna, Bologna, ITA; 2 Department of Biomedical and Neuromotor Sciences (DIBINEM), University of Bologna (Alma Mater Studiorum), Bologna, ITA; 3 Department of Neuroradiology, Maggiore Hospital, IRCCS Istituto delle Scienze Neurologiche di Bologna, Bologna, ITA

**Keywords:** breath-holding index (bhi), carotid artery stenosis, carotid stenting, cerebral vasoreactivity, vasomotor reactivity

## Abstract

While internal carotid artery (ICA) stenosis is typically linked to ischemic stroke, some patients may present with convexity subarachnoid haemorrhage (cSAH). Usually, carotid stenosis leads to decreased vasoreactivity due to chronic hypoperfusion; an exhausted reactivity has been proposed as a causative mechanism for cSAH in these patients. We present a case of cSAH with paradoxically increased vasoreactivity. A 65-year-old male presented with a headache and was diagnosed with bilateral cSAH and severe bilateral ICA stenosis (right near-occlusion, 85% left). MRI showed recent small asymptomatic ischemic lesions in the left hemisphere. Pre-stenting transcranial Doppler (TCD) revealed marked asymmetry in cerebral flow velocities (left > right) and a blunted waveform on the right MCA. Vasoreactivity testing demonstrated bilateral hyperreactivity (breath-holding index (BHI) >2.5). The patient underwent left ICA stenting, resulting in restored symmetry of flow velocities and normalisation of vasoreactivity indices. This case highlights the complex interplay between cerebral autoregulation, vasoreactivity, and stroke and cSAH risk in carotid disease. The normalisation of vasoreactivity post-stenting suggests that pre-existing hyperreactivity was a compensatory response to chronic hypoperfusion. Hyperreactivity may have favoured the cSAH. Vasoreactivity testing provided valuable insights into cerebrovascular compensation and treatment response.

## Introduction

Convexity subarachnoid haemorrhage (cSAH) is an uncommon and diagnostically challenging condition, often linked to hemodynamic stress, impaired autoregulation, and cerebrovascular instability. While cSAH is frequently associated with conditions such as cerebral amyloid angiopathy and reversible cerebral vasoconstriction syndrome, its occurrence in patients with high-grade internal carotid artery (ICA) stenosis raises important questions about the role of chronic hypoperfusion and the compensatory responses of cerebral circulation [1]. Traditionally, severe ICA stenosis is associated with diminished vasoreactivity due to an exhausted autoregulatory capacity [2]. The maximal arteriolar vasodilatation has been linked to increased vascular frailty. Unexpectedly, we discovered a case of cSAH with vasomotor hyperreactivity (informed consent was obtained from the patient).

## Case presentation

**Figure 1:**
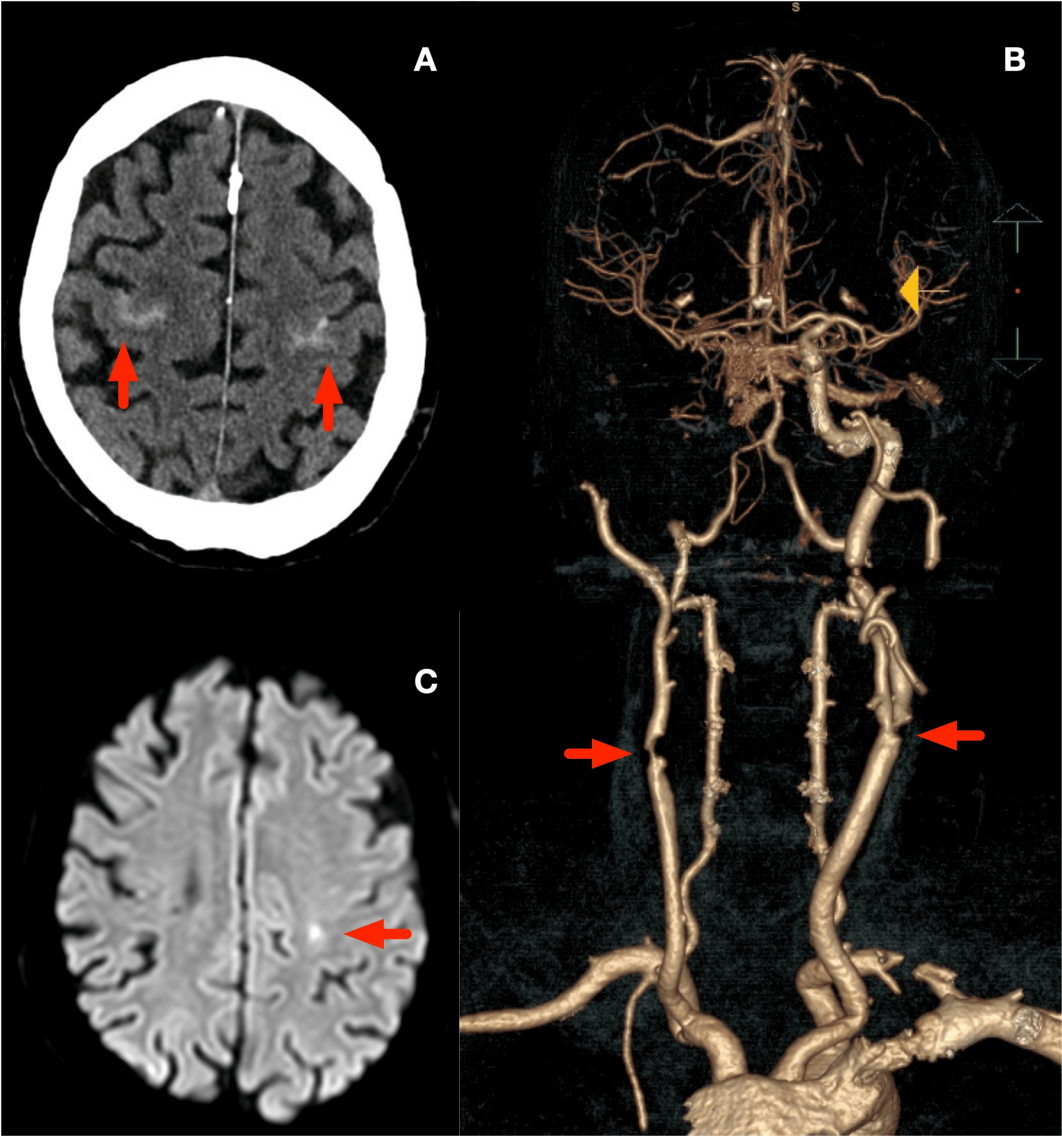
Neuroradiological findings. A: Non-contrast CT scan (NCCT) showing bilateral convexity subarachnoid haemorrhage (cSAH) (red arrows). B: Angio-CT volume rendering reconstruction, showing right internal carotid artery (ICA) steno-occlusion and left ICA stenosis (red arrows). C: Diffusion-weighted magnetic resonance (DWI-MR) showing left corona radiata acute ischemic lesion (red arrow).

A 65-year-old male with a history of hypertension, hypercholesterolemia, and a previous right fronto-insular ischemic stroke presented with a sudden onset of a severe headache and transient left-sided weakness. His neurological symptoms resolved within 24 hours. CT scan identified bilateral cSAH (Figure 1A). CT angiography (CTA) and carotid ultrasound showed a right ICA near-occlusion and hemodynamic stenosis (NASCET 85%) in the proximal left ICA (Figure 1B). MRI further demonstrated bilateral cSAH, with a small subacute ischemic lesion in the left hemisphere (Figure 1C) and chronic ischemic changes in the right hemisphere.

A robotic transcranial Doppler (TCD) study was conducted to assess cerebral vasoreactivity. Thirty seconds of breath-holding and hyperventilation were performed during monitoring of bilateral middle cerebral artery velocity and EtCO_2_. Pre-stenting evaluations revealed a significant asymmetry in flow velocities between hemispheres. Baseline mean flow velocity (MFV) in the left middle cerebral artery was 46.75 cm/s, whereas the right side exhibited markedly lower values at 28.18 cm/s. Moreover, the flow morphology in the right middle cerebral artery (MCA) appeared blunted, a characteristic pattern suggestive of the hemodynamic impact of subocclusive stenosis.

**Figure 2:**
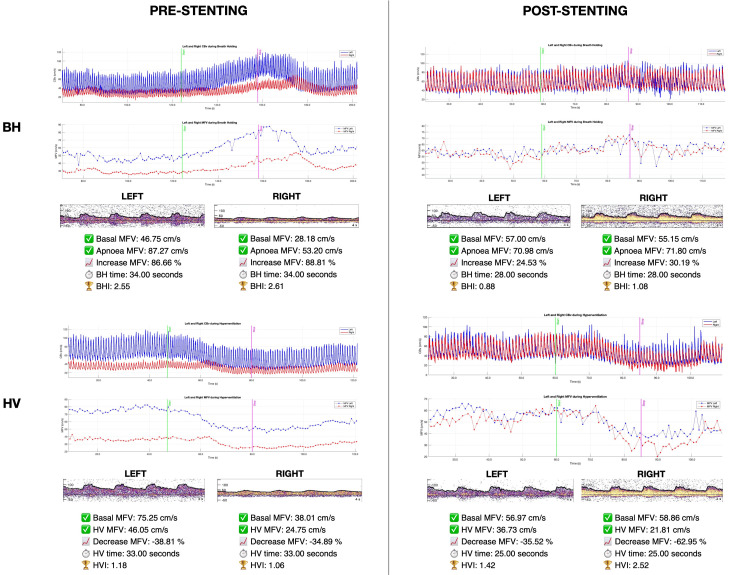
Cerebrovascular reactivity (CVR) to breath-holding (BH, top panels) and hyperventilation (HV, bottom panels) before (left) and after (right) carotid artery stenting. For each condition, the upper plots display raw bilateral Doppler signals (left middle cerebral artery (MCA): blue; right MCA: red), the middle plots show the derived trends of mean flow velocity (MFV) over time for both sides, and the lower panels illustrate representative waveform morphology for each hemisphere.

Surprisingly, the apnoea test demonstrated an exaggerated vasodilatory response, with MFV increasing by 86.66% on the left and 88.81% on the right, yielding breath-holding indices (BHIs) of 2.55 and 2.61, respectively, indicative of marked vasomotor hyperreactivity. Interestingly, the right MCA showed a significant latency reaching the peak of flow velocity increase compared to the contralateral side (Figure 2). Conversely, hyperpnea testing revealed a corresponding vasoconstrictive response, with MFV reductions of 38.81% on the left and 34.89% on the right (hyperventilation index (HVI): 1.18 on the left side, 1.06 on the right side).

A multidisciplinary discussion concluded that, despite the greater degree of stenosis in the right ICA, the presence of ischemic lesions in the left hemisphere justified prioritising left ICA revascularisation. Dual antiplatelet therapy was initiated three days prior to left ICA stenting. The procedure was successfully performed on the 14th day after admission, restoring normal flow patterns in the treated vessel without periprocedural complications. Post-stenting evaluations revealed a remarkable transformation in cerebrovascular hemodynamics. Baseline MFV became symmetrical, with values of 57.00 cm/s on the left and 55.15 cm/s on the right, suggesting improved hemispheric perfusion balance. The previously exaggerated BHI values normalised bilaterally to 0.88 on the left and 1.08 on the right, indicating a restoration of physiological vasoreactivity. Furthermore, the spectral waveform analysis demonstrated that the post-stenotic flow abnormalities observed in the right ICA prior to intervention were no longer present, suggesting that left-sided revascularisation contributed to an overall stabilisation of intracranial hemodynamics.

Despite the initial presentation with cSAH, no worsening of haemorrhage or new bleedings were demonstrated after dual antiplatelet therapy start nor after stenting, even in follow-up imaging.

## Discussion

This case highlights the dynamic nature of cerebrovascular adaptation in patients with severe bilateral ICA stenosis and cSAH. cSAH is a rare but already described finding in patients with ICA stenosis and supposed vasomotor dysregulation. Usually, these patients already have maximally dilated cerebral arterioles to maintain baseline cerebral blood flow (CBF) and may not be able to augment their CBF further in response to challenges, like breath-holding [3]. Our case displayed a completely different situation: before stenting, the patient exhibited an exaggerated vasoreactive response. In this setting, the cSAH could result from excessive CBF increase in either abnormally dilatated pial collaterals (which usually present little to no myogenic response, in distinction to terminal arterioles) [4] or frail newly formed collateral vessels [1]. In our case, a slight hypertrophy of cortical branches was angiographically demonstrated only on the right side, but not on the left.

Following revascularisation, cerebrovascular function underwent substantial normalisation. The restoration of symmetrical flow velocities across both hemispheres suggested improved global cerebral perfusion. Most notably, the BHI values, which were markedly elevated pre-stenting, returned to physiologic ranges, indicating that the exaggerated compensatory reactivity had subsided.

Interestingly, the response to hypercapnia (BHI) pre-stenting was more exaggerated than the response to hypocapnia (HVI), suggesting a nonlinear relationship between CO_2_ levels and cerebrovascular reactivity in vessels chronically exposed to hypoperfusion.

The stenting procedure did not determine worsening of cSAH or hyperperfusion syndrome. For the former, restoring a normal bilateral vasomotor response could have contributed to protecting against bleeding. Regarding the latter, the preserved pre-stent vasoconstrictive response to hyperventilation is a potential indicator of active buffering in the event of a steep increase in flow [5].

This work has some limitations. This report describes a single case, which inherently limits the generalizability of our findings. In addition, cerebrovascular reactivity was assessed using transcranial Doppler-based breath-holding and hyperventilation tests, which, although validated and non-invasive, provide indirect measures of cerebrovascular reserve and are susceptible to interindividual variability and CO₂ response thresholds. Lastly, while the temporal association between revascularisation and hemodynamic normalisation suggests a causal relationship, this cannot be definitively established in the absence of a controlled experimental framework. Future prospective studies are needed to validate the hypothesis that paradoxical vasomotor hyperreactivity may contribute to convexity subarachnoid haemorrhage in patients with carotid stenosis.

## Conclusions

This report highlights, for the first time to our knowledge, a potential link between excessive vasoreactivity in the setting of bilateral ICA stenosis and the occurrence of cSAH. The observation supports the hypothesis that an exaggerated vasodilatation response in dilated pial collaterals or newly formed collateral vessels, under conditions of chronic hemodynamic stress, may play a role in the pathogenesis of cSAH. After stenting, as a result of the improved hemodynamic condition, reactivity normalized, and cSAH did not worsen even with dual antiplatelet therapy. On the other hand, the preserved response to hypercapnia is a potential indicator of protection from hyperperfusion syndrome.

More broadly, our findings emphasize the clinical relevance of vasomotor reactivity testing in patients with carotid stenosis, not only to better understand the hemodynamic consequences of arterial narrowing, but also to identify possible associations with cerebrovascular complications, such as cSAH. Vasomotor reactivity testing may also inform about the potential impact of revascularization therapies.
